# Occurrence of a novel mastrevirus in sugarcane germplasm collections in Florida, Guadeloupe and Réunion

**DOI:** 10.1186/s12985-017-0810-9

**Published:** 2017-07-28

**Authors:** Wardatou Boukari, Ricardo I. Alcalá-Briseño, Simona Kraberger, Emmanuel Fernandez, Denis Filloux, Jean-Heinrich Daugrois, Jack C. Comstock, Jean-Michel Lett, Darren P. Martin, Arvind Varsani, Philippe Roumagnac, Jane E. Polston, Philippe C. Rott

**Affiliations:** 1 IFAS, Everglades Research & Education Center, University of Florida, Belle Glade, FL 33430 USA; 2 IFAS, Plant pathology Department, University of Florida, Gainesville, FL 32611 USA; 3 The Biodesign Center for Fundamental and Applied Microbiomics, Center for Evolution and Medicine, School of Life Sciences, Arizona State University, Tempe, AZ 85287-5001 USA; 4 CIRAD-INRA-Montpellier SupAgro, UMR BGPI, Campus International de Baillarguet, 34398 Montpellier, France; 5 USDA-ARS, Sugarcane Field Station, Canal Point, FL 33438 USA; 6 CIRAD, UMR PVBMT, Pôle de Protection des Plantes, 7 chemin de l’IRAT, 97410 Saint-Pierre, Ile de la Réunion France; 7 Computational Biology Group, Institute of Infectious Disease and Molecular Medicine, University of Cape Town, Faculty of Health Sciences, Observatory, Cape Town, 7925 South Africa; 8 Structural Biology Research Unit, Department of Clinical Laboratory Sciences, University of Cape Town, Cape Town, 7001 South Africa

**Keywords:** *Geminiviridae*, Mastrevirus, *Saccharum* spp., Sugarcane, Sugarcane striate virus

## Abstract

**Background:**

In Africa and Asia, sugarcane is the host of at least seven different virus species in the genus *Mastrevirus* of the family *Geminiviridae*. However, with the exception of *Sugarcane white streak virus* in Barbados, no other sugarcane-infecting mastrevirus has been reported in the New World. Conservation and exchange of sugarcane germplasm using stalk cuttings facilitates the spread of sugarcane-infecting viruses.

**Methods:**

A virion-associated nucleic acids (VANA)-based metagenomics approach was used to detect mastrevirus sequences in 717 sugarcane samples from Florida (USA), Guadeloupe (French West Indies), and Réunion (Mascarene Islands). Contig assembly was performed using CAP3 and sequence searches using BLASTn and BLASTx. Mastrevirus full genomes were enriched from total DNA by rolling circle amplification, cloned and sequenced. Nucleotide and amino acid sequence identities were determined using SDT v1.2. Phylogenetic analyses were conducted using MEGA6 and PHYML3.

**Results:**

We identified a new sugarcane-infecting mastrevirus in six plants sampled from germplasm collections in Florida and Guadeloupe. Full genome sequences were determined and analyzed for three virus isolates from Florida, and three from Guadeloupe. These six genomes share >88% genome-wide pairwise identity with one another and between 89 and 97% identity with a recently identified mastrevirus (KR150789) from a sugarcane plant sampled in China. Sequences similar to these were also identified in sugarcane plants in Réunion.

**Conclusions:**

As these virus isolates share <64% genome-wide identity with all other known mastreviruses, we propose classifying them within a new mastrevirus species named Sugarcane striate virus. This is the first report of sugarcane striate virus (SCStV) in the Western Hemisphere, a virus that most likely originated in Asia. The distribution, vector, and impact of SCStV on sugarcane production remains to be determined.

**Electronic supplementary material:**

The online version of this article (doi:10.1186/s12985-017-0810-9) contains supplementary material, which is available to authorized users.

## Background

Sugarcane (interspecific hybrids of *Saccharum* spp.) is an economically important perennial crop grown mainly for sugar and ethanol production in the tropical and sub-tropical regions of the world. Sugarcane is not native to the vast majority of countries where it is commercially grown. Introduction of sugarcane clones is therefore essential to establish sugarcane industries or for breeding purposes. In a production area, sugarcane clones are vegetatively propagated via stalk pieces (cuttings) and this planting material is also used for movement of germplasm between geographical locations. However, this material can be infected by various pathogens [1]. Nowadays, quarantine stations that employ robust diagnostic techniques are used for exchange of sugarcane germplasm [2]. This was not the case several decades ago or during the nineteenth century when *S. officinarum* clones were collected, especially in New Guinea (Melanesia) which is generally accepted as a center of diversity for the genus *Saccharum* [3]. Additionally, symptomless plants infected with unknown viral pathogens may still escape quarantine procedures when adequate diagnostic methods are not available.

Sugarcane can be infected by viruses belonging to at least seven different virus species in the genus *Mastrevirus* of the family *Geminiviridae*: *Sugarcane streak virus* [4], *Sugarcane streak Egypt virus* [5], *Sugarcane streak Reunion virus* [6], *Sugarcane white streak virus* [7], *Maize streak virus* [8], *Saccharum streak virus* [9], and Sugarcane chlorotic streak virus [10]. Strikingly, with the exception of sugarcane white streak virus (SCWSV), six of these viruses have natural geographical ranges that are apparently restricted to Africa and the Indian Ocean islands off the African coast. Although most sugarcane-infecting mastreviruses are primarily found in Africa, they may appear less prevalent elsewhere in the world simply because less effort has been expended to detect them outside Africa.

In this regard, the use of high throughput sequencing based approaches and/or rolling circle amplification (RCA) could enable a more balanced global search for sugarcane-infecting mastreviruses. These techniques coupled with full-genome cloning and sequencing have recently proved useful in identifying unknown mastreviruses within both sugarcane field and quarantine contexts [7, 10], and have revealed the presence of mastreviruses in parts of the world where they were previously unknown [7, 11, 12].

## Methods

Leaf samples from different species of *Saccharum* and related genera were collected from the Biological Resource Centre for Tropical Plants in Guadeloupe in 2013 (*n* = 300), from commercial fields in the Everglades Agricultural Area (Belle Glade and Clewiston, FL) in 2013/2014 (*n* = 95), the germplasm collection of the USDA-ARS (Miami, FL) in 2013/2014 (*n* = 113), and from CIRAD’s germplasm collection in Réunion Island in 2014 (*n* = 209) (Table 1). This sampling was part of a project whose goal was to catalogue sugarcane-infecting virus species and characterize viral communities within sugarcane plants. These leaf samples represent plants that originated (source of first collection or source of sugarcane hybrid creation) from at least 36 different geographical locations (source of >50 clones unknown; Table 2). Freshly collected leaf pieces taken from the top visible dewlap leaf were sealed in plastic bags or dried on CaCl_2_ and then shipped to CIRAD in Montpellier, France where all samples were further processed. A virion-associated nucleic acids (VANA)-based metagenomics approach was used to analyze the viromes of each of the 717 plant samples from these four sites, as described by Palanga et al. [13]. Briefly, total nucleic acids were extracted from homogenized leaf tissue and used to produce single strand cDNA libraries. Double strand cDNA libraries were produced by klenow polymerization and amplified by PCR. Tagged DNA amplicons from 96 samples were pooled and sequenced using a 454 sequencing plate (Beckman Coulters Genomics, USA).

**Table 1:** *Saccharum* species and sugarcane related species sampled in this study

Plant species	Number of clones sampled in the sugarcane germplasm collection in			Number of sugarcane clones sampled in commercial fields in Florida
	Guadeloupe	Réunion	Florida	
*Erianthus arundinaceus*	1	4	0	0
*Erianthus spunknow*	0	0	2	0
*Miscanthus floridulus*	0	0	1	0
*Saccharum barberi*	1	1	1	0
*Saccharum edule*	1	0	1	0
*Saccharum officinarum*	18	3	80	0
*Saccharum robustum*	10	1	2	0
*Saccharum sinense*	3	3	2	0
*Saccharum spontaneum*	14	0	9	0
*Saccharum* spp. (hybrid)	252	197	14	33
*Sorghum plumosum*	0	0	1	0
Total	300	209	113	33

**Table 2:** Original source of the *Saccharum* species and sugarcane related species sampled in this study

Original source	Number of plants sampled in the sugarcane germplasm collection in			Number of sugarcane plants sampled in commercial fields in Florida
	Guadeloupe	Réunion	Florida	
Argentina	6	2	0	0
Australia	15	17	1	0
Barbados	31	12	0	0
Belize	5	0	0	0
Brazil	9	15	0	0
China	4	0	1	0
Colombia	3	0	0	0
Cuba	10	1	0	0
Dominican Republic	15	0	0	0
Fiji	15	9	6	0
Florida	11	10	13	85
Guadeloupe	15	5	0	0
Guyana	9	2	0	0
Hawaii	3	14	3	0
India	26	12	4	0
Indonesia	5	0	1	0
Indonesia-Java	8	4	8	4
Indonesia-Kalimantan	2	2	3	0
Iran	0	0	1	0
Jamaica	7	0	0	0
Japan	0	1	0	0
Malaysia	5	0	1	0
Mauritius	7	21	0	0
Mexico	8	4	0	0
Myanmar	0	0	1	0
New Guinea	12	3	27	2
Pakistan	3	0	0	0
Philippines	6	0	3	0
Puerto Rico	2	3	0	0
Réunion	16	47	0	0
Saipan	1	1	0	0
Saudi Arabia	0	0	1	0
South Africa	9	10	1	0
Sudan	7	1	0	0
Taiwan	11	7	1	4
Trinidad	5	0	0	0
Vanuatu (New Hebrides)	0	0	1	0
Unknown	14	6	36	0
Total	300	209	113	95

One plant sampled per clone in each of the three germplasm collections. Samples of commercial fields in Florida include 32 plants (16 clones) of former commercial sugarcane varieties maintained in commercial field environment

Following de novo contig assembly of cleaned reads performed using CAP3 [14] and searches using BLASTn (Basic Local Alignment Search Tool) and BLASTx [15], 34 contigs with detectable homology to mastreviruses were identified from nine of these 717 plant samples (four from Florida, three from Guadeloupe, and two from Réunion). BLASTx searches revealed that these contigs potentially encode proteins from four different mastrevirus-like genes (V1, V2, C1 and C2) that were between 86 and 100% identical to those encoded by the genome of a sugarcane-infecting mastrevirus from China that was deposited in GenBank in September 2015 (isolate WZG, GenBank accession number KR150789). It is noteworthy that this virus genome from China was identified by its depositor (Wen and collaborators, Guangxi University, China) as a sugarcane streak virus isolate, despite the fact that at the time it shared less than 64% pairwise genome sequence identity with any other known mastrevirus and should therefore have been identified as a novel species.

**Fig. 1:**
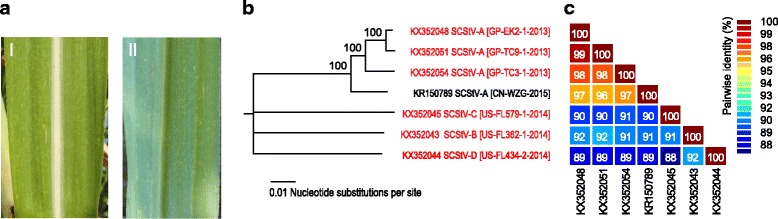
**a**. A leaf fragment exhibiting yellow striation symptoms from a *Saccharum officinarum* plant (variety NG28-020) found to be infected by sugar cane striate virus. I: Upper leaf surface, II: Lower leaf surface. **b**. A maximum likelihood phylogenetic tree constructed from the full genome sequences of six SCStV isolates that were determined in this study, together with a SCStV isolate from China (inferred using GTR + I + G substitution model which was selected as the best fitting model using jModelTest [19]. Support for branches was tested using an approximate likelihood ratio test (aLRT). Branches with less than 80% support have been collapsed. **c**. Pairwise identity matrix of the SCStV genome sequences

Only one of the nine plants within which mastrevirus sequences were detected (a *S. officinarum* from the Miami germplasm collection, NG28-020) displayed any discernable chlorotic streak- or striation-like symptoms such as those caused by many of the known monocotyledonous plant-infecting mastreviruses (Fig. 1). Total DNA was extracted from the samples from Florida/Miami (three plants of *Saccharum spontaneum* Iranspon, one of *S. barberi* Ketari, one of *S. officinarum* NG28-020, and one from *S. officinarum* Pundia), Guadeloupe (one plant of noble cane, *S. officinarum* EK2, and one plant each of commercial sugarcane cultivars TC3 and TC9), and Réunion (one plant of *S. barberi* Sararoo 1492 and one plant from *S. sinense* UBA Aust). Total DNA was enriched for mastrevirus full genomes by rolling circle amplification (RCA) using Phi29 DNA polymerase (TempliPhi™, GE Healthcare, USA) as previously described by Shepherd et al. [16]. The RCA products were either used as templates for polymerase chain reaction (PCR)-based amplification using a set of primers designed based on the VANA contigs (Table 3), or were restricted using either *Bam*HI or *Pst*I. The amplified products were ligated to pJET1.2 (Thermo Fisher USA), whereas the restricted ~2.8Kb mastrevirus genome-length fragments were ligated to pBlueScript (Agilent, USA). The resulting recombinant plasmids were Sanger sequenced by primer walking at Beckman Coulter Genomics (plasmids from the Florida/Miami site) and Macrogen Inc. (plasmids from the Guadeloupe site).

**Table 3:** Characteristics of the full genome sequences of the new Mastrevirus isolates recovered from plants collected in Florida and Guadeloupe

GenBank accession #	Strain	Isolate	Host	Original source	Sampling location	Collection date	Sequence length (nt)	Restriction/primers
KX352041	D	FL_30-1	*Saccharum spontaneum* (Iranspon)	Iran	USA (Florida)	30 Sept. 2014	2747	*Pst*I
KX352042	D	FL_30-2	*Saccharum spontaneum* (Iranspon)	Iran	USA (Florida)	30 Sept. 2014	2747	*Pst*I
KX352044	**D**	**FL_434-2**	*Saccharum spontaneum* (Iranspon)	Iran	USA (Florida)	30 Sept. 2014	2743	*Pst*I
KX352047	D	FL_897-1	*Saccharum spontaneum* (Iranspon)	Iran	USA (Florida)	30 Sept. 2014	2742	*Pst*I
KX352045	**C**	**FL_579-1**	*Saccharum barberi* (Ketari)	India	USA (Florida)	30 Sept. 2014	2740	*Bam*HI
KX352046	C	FL_579-2	*Saccharum barberi* (Ketari)	India	USA (Florida)	30 Sept. 2014	2746	*Bam*HI
KX352040	C	FL_579-3	*Saccharum barberi* (Ketari)	India	USA (Florida)	30 Sept. 2014	2738	*Bam*HI
KX352043	**B**	**FL_362-1**	*Saccharum officinarum* (NG28-020)	New Guinea	USA (Florida)	30 Sept. 2014	2749	*Bam*HI
KX352048	**A**	**GP_EK2-1**	*Saccharum officinarum* (EK2)	Indonesia	Guadeloupe	11 Dec. 2013	2747	F-5′-CCGAACTGAATGGAAAAATACAACCGGAGG-3′ R-5′-ACCCCCAGACGCTTCACGAACTTC-3’
KX352056	A	GP_EK2-2	*Saccharum officinarum* (EK2)	Indonesia	Guadeloupe	11 Dec. 2013	2741	F-5’-CCGAACTGAATGGAAAAATACAACCGGAGG-3′ R-5′-ACCCCCAGACGCTTCACGAACTTC-3’
KX352049	A	GP_EK2-3	*Saccharum officinarum* (EK2)	Indonesia	Guadeloupe	11 Dec. 2013	2747	F-5’-CCGAACTGAATGGAAAAATACAACCGGAGG-3′ R-5′-ACCCCCAGACGCTTCACGAACTTC-3’
KX352050	A	GP_EK2-4	*Saccharum officinarum* (EK2)	Indonesia	Guadeloupe	11 Dec. 2013	2747	F-5’-CCGAACTGAATGGAAAAATACAACCGGAGG-3′ R-5′-ACCCCCAGACGCTTCACGAACTTC-3’
KX352054	**A**	**GP_TC3-1**	Sugarcane (TC3)	Malaysia	Guadeloupe	11 Dec. 2013	2749	F-5’-CCGAACTGAATGGAAAAATACAACCGGAGG-3′ R-5′-ACCCCCAGACGCTTCACGAACTTC-3’
KX352055	A	GP_TC3-2	Sugarcane (TC3)	Malaysia	Guadeloupe	11 Dec. 2013	2749	F-5’-CCGAACTGAATGGAAAAATACAACCGGAGG-3′ R-5′-ACCCCCAGACGCTTCACGAACTTC-3’
KX352051	**A**	**GP_TC9-1**	Sugarcane (TC9)	Malaysia	Guadeloupe	11 Dec. 2013	2747	F-5’-CCGAACTGAATGGAAAAATACAACCGGAGG-3′ R-5′-ACCCCCAGACGCTTCACGAACTTC-3’
KX352052	A	GP_TC9-2	Sugarcane (TC9)	Malaysia	Guadeloupe	11 Dec. 2013	2747	F-5’-CCGAACTGAATGGAAAAATACAACCGGAGG-3′ R-5′-ACCCCCAGACGCTTCACGAACTTC-3’
KX352053	A	GP_TC9-3	Sugarcane (TC9)	Malaysia	Guadeloupe	11 Dec. 2013	2747	F-5’-CCGAACTGAATGGAAAAATACAACCGGAGG-3′ R-5′-ACCCCCAGACGCTTCACGAACTTC-3’

Isolates that differ only by the last number originated from the same host plant; isolates selected for phylogenetic analyses are in bold

## Results

Seventeen genome sequences (2738-2749 nt) were obtained using either RCA followed by enzymatic restriction, or PCR using back-to-back primers, followed by cloning of full length restricted fragments or full length amplicons and Sanger sequencing (Table 3). Three full genome sequences were obtained from a single *S. barberi* plant (FL_579-1, −2 and −3), five from two different *S. officinarum* plants (FL_362-1, GP_EK2-1, −2, −3 and −4), four from three different *S. spontaneum* plants (FL_30-1, −2, FL_434-2 and FL_897-1) and five from two different sugarcane hybrids (GP_TC3-1, −2, GP_TC9-1, −2 and −3; Table 3). RCA amplification products were obtained for three additional infected host plants: *S. officinarum* Pundia from the USA collection, and *S. barberi* Sararoo 1492 and *S. sinense* UBA Aust from the collection in Réunion. However, cloning of these amplicons was unsuccessful after several attempts and for unknown reasons. BLASTn comparisons between the 17 genome sequences and those in GenBank indicated that they were all most similar (89 to 97% identity) to the novel sugarcane-infecting mastrevirus genome sequence from China deposited under GenBank accession number KR150789.

One representative virus isolate was selected for each of the six host plants that yielded entire genome sequences: FL_434-2 (*S. spontaneum* Iranspon), FL_579-1 (*S. barberi* Ketari), FL_362-1 (*S. officinarum* NG28-020), GP_EK2-1 (*S. officinarum* EK2), GP_TC3-1 (*Saccharum* hybrid TC3), and GP_TC9-1 (*Saccharum* hybrid TC9). These six new sugarcane-infecting mastrevirus sequences were aligned together with the sugarcane mastrevirus sequence from China and a representative set of mastrevirus genome sequences that were available in GenBank in January 2017. Whole-genome pairwise nucleotide sequence identities, and replication associated protein (Rep) and capsid protein (CP) pairwise amino acid sequence identities were determined using SDT v1.2 [17]. The six new mastreviruses isolates and their close relative from China share <64% genome-wide identity with other known mastreviruses and thus, based on the International Committee for Virus Taxonomy endorsed mastrevirus species demarcation threshold of 78% genome-wide identity, these virus isolates should all be classified as belonging to a new species: one which we propose be named Sugarcane striate virus based on the symptoms observed on one of the infected *S. officinarum* plants from which one of the new genomes was isolated (Fig. 1). It is noteworthy that no other RNA or DNA virus sequence was found in this plant using the metagenomics approach described above, thus providing strong evidence for the association between the striation-like symptoms and the new mastrevirus.

Based on the accepted mastrevirus strain demarcation threshold of 94% genome-wide nucleotide sequence identity [18], the six sugarcane striate virus (SCStV) isolates were further classified into four different strains named A, B, C and D (Fig. 1). The three isolates from Guadeloupe and the isolate from China (isolated from *S. officinarum* and *Saccharum* interspecific hybrids) all belong to strain A, whereas the other three strains are comprised of the three isolates from Florida. These latter three strains were each associated with a specific species of *Saccharum*: the strain B isolate (FL_362-1) was found in a *S. officinarum* plant, the strain C isolate (FL_579-1) was found in a *S. barberi* plant and the strain D isolate (FL_434-2) was found in a plant of *S. spontaneum* variety Iranspon. A maximum-likelihood phylogenetic tree (constructed with the best fitting model, GTR + G + I using jModelTest [19]) based on the full genome alignment also supports the proposed strain classification (Fig. 1).

Furthermore, four contigs from each of two sugarcane plants from Réunion Island (*S. barberi* Sararoo 1492 and *S. sinense* UBA_Aust, both originating from India) ranging in size from between 171 and 1214 nucleotides were obtained after de novo assembly from the VANA reads. These contigs corresponded to mastrevirus V1, V2 and C1 open reading frames that were between 93 and 100% identical to the homologous regions of the SCStV isolate from China, WZG, and 99% identical to the homologous regions of the three isolates from Guadeloupe. The presence of SCStV in the two samples from Réunion was validated by PCR with primers developed using the high throughput sequencing data (data not shown). We can therefore conclude that isolates of SCStV are also present within the sugarcane collection held by CIRAD on Réunion Island.

**Fig. 2:**
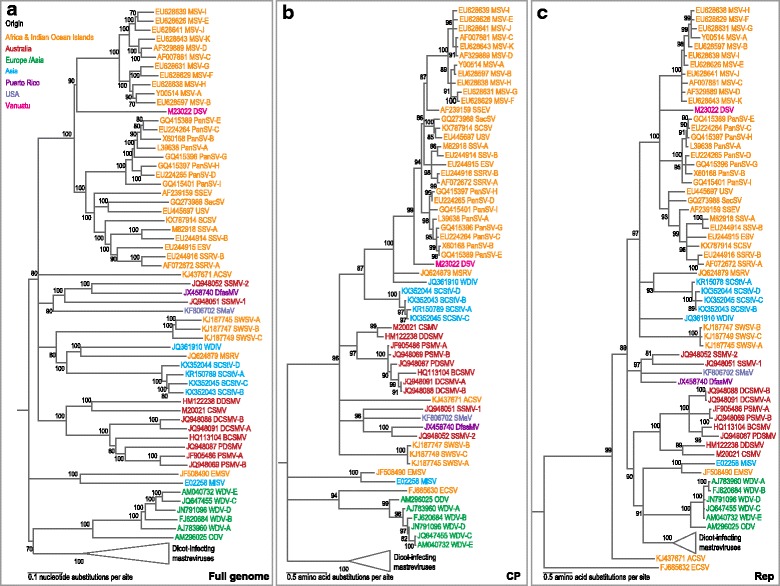
**a**. Neighbor joining phylogenetic tree (inferred using the Jukes-Cantor nucleotide substitution model) of representative SCStV isolates together with a representative selection of mastrevirus genomes. Numbers associated with branches indicate the percentage of 1000 bootstrap replicates that support the existence of these branches. Branches with <60% bootstrap support have been collapsed. Maximum likelihood phylogenetic trees of the inferred CP (**b**) and Rep (**c**) amino acid sequences of the same genomes as in A inferred using LG + G + I substitution model (selected as the best fit using ProtTest [23]. Numbers associated with branches indicate the percentage probability that the branches exist according to an approximate likelihood ratio test (aLRT) of branch support. Branches with <80% aLRT support have been collapsed. The CP and Rep phylogenetic trees were rooted with the CP and Rep sequences of beet curtly top virus (BCTIV), a member of the genus *Becurtovirus* of the family *Geminiviridae*

A neighbor-joining phylogenetic tree was inferred from the aligned sequences of one of each of the four SCStV strains together with 69 other representative mastrevirus genomes using MEGA6 [20] (Fig. 2). Additionally, maximum-likelihood phylogenetic trees were constructed from the inferred CP and Rep amino acid sequences encoded by these mastrevirus genomes with PHYML3 [21] (Fig. 2). In all three trees, the SCStV sequences cluster with other monocotyledonous plant-infecting mastreviruses. The SCStV genomes are most closely related to maize streak Reunion virus (MSRV) and wheat dwarf India virus (WDIV) with which they share between 63 and 64% pairwise genome sequence identity (Additional file 1). The SCStV CP and Rep amino acid sequences respectively share <50% and <56% identity with those of other mastreviruses (Additional files 2 and 3).

## Discussion

Movement of sugarcane germplasm using stalk cuttings greatly facilitates the spread of sugarcane viruses, especially unknown viruses for which no detection methods are available or which can escape quarantine facilities in asymptomatic plants. SCStV was found in four different species of *Saccharum* (*S. officinarum*, *S. barberi*, *S. spontaneum*, and *S. sinense*) and only in two commercial sugarcane hybrids (TC3 and TC9) although over 400 hybrid clones (*Saccharum* spp.) were tested (Table 1). This suggests that the virus occurs mainly in botanical species that were collected since the late 1800s [3]. However, it cannot be excluded that the virus occurs and is actively spread in locations that were not sampled herein as sugarcane is grown in more than 100 locations or countries around the world [22].

The two commercial sugarcane varieties which were found infected by SCStV herein have been created in Malaysia. With the exception of sugarcane bacilliform virus (a virus that can integrate the sugarcane genome), no sugarcane virus has been reported as seed (also called fuzz) transmitted [1]. Assuming that SCStV is not seed transmitted either, cultivars TC3 and TC9 have been infected by this virus in Malaysia, and the insect vector(s) (which is presumably a leafhopper) should be present at least in this geographical location. The vector(s) might also be present in China where isolate WZG was also collected from sugarcane (identity and source unknown).

It is clear from our study that SCStV has a broader geographical distribution than any known mastrevirus species. Although its nearest relatives have been found in India (WDIV), Africa and Réunion Island (MSRV), both of these species are distantly related enough to SCStV that they provide little resolution with respect to where SCStV may have originated (other than probably somewhere in the eastern hemisphere). However, plants infected by SCStV in this study were all originally sourced from Asian countries (India, Indonesia, Iran, Malaysia, New Guinea), thus suggesting an Asian origin for SCStV. In this regard, it is of some concern that this virus is presently resident within at least two locations in the western hemisphere (USA and Guadeloupe). It is noteworthy that the SCStV isolates from the USA were identified in plants from three varieties that were introduced to the world germplasm collection in Miami, Florida more than six decades ago. Up until now, SCStV has not been identified in commercially grown sugarcane in Florida, possibly because its insect vector(s) does not occur naturally in Florida.

## Conclusion

SCStV is a newly reported sugarcane virus whose biology is unknown. It would be of great interest therefore to determine (1) the natural vector(s) of SCStV; (2) the distribution of this virus and its vector across the USA, Central America and the Caribbean, (3) the prevalence of the different SCStV strains that have so far been identified in the USA; and (4) the impact of SCStV on sugarcane growth and yields. All of this information will be crucial to assess the economic risks that are posed by SCStV.

## Additional files

Additional file 1: Table S1. Whole genome pairwise comparison. (XLSX 25 kb)

Additional file 2: Table S2. CP gene amino acid sequence pairwise comparison. (XLSX 35 kb)

Additional file 3: Table S3. Rep gene amino acid sequence pairwise comparison. (XLSX 35 kb)

## Abbreviations

CP: Capsid protein
MSRV: Maize streak Reunion virus
PCR: Polymerase chain reaction
RCA: Rolling circle amplification
Rep: Replication associated protein
SCStV: Sugarcane striate virus
SCWSV: Sugarcane white streak virus
VANA: Virion-associated nucleic acids
WDIV: Wheat dwarf India virus
